# Systemic Endotoxemia, Inflammatory Activation, and Lipid Dysregulation in Parkinson’s Disease: Evidence from Circulating LPS-Related Biomarkers and Plasma Lipids

**DOI:** 10.3390/ijms27093711

**Published:** 2026-04-22

**Authors:** Matteo Della Porta, Michela Barichella, Gianni Pezzoli, Roberta Cazzola

**Affiliations:** 1 Department of Biomedical & Clinical Sciences, Università di Milano, 20157 Milan, Italy; matteodellaporta92@gmail.com; 2 Fondazione Pezzoli per la Malattia di Parkinson, 20125 Milan, Italy; barichella@parkinson.it (M.B.); pezzoli@parkinson.it (G.P.); 3 ASST Gaetano Pini-CTO, 20126 Milan, Italy

**Keywords:** Parkinson’s disease, endotoxemia, innate immune activation, lipid dysregulation, LPS-related biomarkers

## Abstract

Growing evidence implicates neuroinflammation, gut-derived endotoxemia, and dysregulated lipid metabolism in the pathogenesis of Parkinson’s disease (PD). However, the relationships among circulating lipopolysaccharide (LPS), LPS-handling proteins, systemic inflammatory activation, and lipid fractions remain insufficiently characterized. The aim of this study was to compare LPS levels, LPS-related inflammatory mediators, and plasma lipid parameters between PD patients and matched controls, and to explore correlations among these biomarkers. Twenty PD patients and twenty matched controls underwent fasting venous sampling. Circulating LPS, lipopolysaccharide binding protein (LBP), soluble cluster of differentiation 14 (sCD14), high-sensitivity C-reactive protein (hsCRP), and phospholipid transfer protein (PLTP) were quantified via LAL assay and ELISAs. Serum cholesterol, HDL cholesterol (HDL-C), phospholipids (PLs), HDL-PLs and triacylglycerols (TAGs) were assessed using validated biochemical techniques. LPS concentrations did not differ between groups. However, PD patients showed elevated sCD14 and hsCRP levels, reduced LBP, and increased PLTP. Lipid profiling revealed lower total cholesterol and reduced HDL-associated cholesterol and phospholipids in PD, while TAG levels remained unchanged. Correlation analyses indicated coordinated associations between inflammatory markers and lipid fractions, with distinct interaction patterns in PD compared with controls. These findings support a mechanistic interplay among endotoxemia, innate immune activation, and lipid dysregulation in the pathophysiology of PD.

## 1. Introduction

Parkinson’s disease (PD) is the second most common neurodegenerative disorder and manifests with motor symptoms such as bradykinesia, tremor, and rigidity, together with frequent non-motor features including constipation, sleep disturbances, mood alterations, and autonomic dysfunction [1]. Neuropathologically, PD is defined by dopaminergic neuron loss in the substantia nigra pars compacta and by the accumulation of misfolded α-synuclein (α-Syn) in Lewy bodies [2].

Although the causes of PD are not fully understood, both genetic predisposition and environmental factors contribute to its development. Increasing evidence highlights a central role for immune dysregulation, including chronic microglial activation, elevated pro-inflammatory cytokines, blood–brain barrier alterations, and peripheral immune cell infiltration [1]. Toll-like receptor (TLR) signaling is a key pathway in this process: TLRs are upregulated in PD, and α-Syn itself can act as a damage-associated molecular pattern, promoting microglial activation and inflammatory mediator release [3]. Lipopolysaccharide (LPS), a Gram-negative bacterial endotoxin, is a potent activator of innate immunity and has been linked to PD-related inflammation [4,5]. PD monocytes show heightened sensitivity to LPS [6], and TLR4 expression is increased in immune cells from blood and gut, suggesting a primed inflammatory state [7]. Gut–brain axis dysfunction—including constipation, dysbiosis, and pathological changes in the gut—may further enhance exposure to bacterial products such as LPS [8,9,10,11].

Key factors regulating the inflammatory response to endotoxin exposure include lipopolysaccharide-binding protein (LBP), cluster of differentiation (CD) 14, phospholipid transfer protein (PLTP), and plasma lipoproteins. LBP promotes LPS delivery to CD14 and TLR4 signaling [12,13], while PLTP mainly contributes to LPS neutralization [14,15]. Both LBP and PLTP can extract LPS from bacterial membranes and transfer it to high-density lipoprotein (HDL), resulting in the functional neutralization of LPS [15].

Recent evidence suggests a relationship between serum lipid profiles and PD, with HDL-cholesterol correlating with neurodegenerative biomarkers [16]. Phospholipids (PLs), the main lipid component of lipoprotein surfaces, are key determinants of LPS–lipoprotein binding [13]. Changes in lipid profiles observed in PD, including alterations in phospholipid species [17], may hinder the effectiveness of LPS detoxification, particularly due to changes in the quantity or composition of HDL. These modifications could ultimately reduce the efficiency of LPS detoxification.

Overall, data from immunology, lipid metabolism, and neuropathology suggest that endotoxemia, immune activation, lipid dysregulation, and α-Syn biology are interconnected in PD. This study investigates plasma levels of LPS-related inflammatory markers (LPS, LBP, sCD14, and high-sensitivity C-reactive protein [hsCRP]) and lipid parameters (cholesterol fractions, PLs, and HDL composition) in PD versus controls to clarify their mechanistic relationships.

## 2. Results

Population characteristics showed no significant differences in age, body mass index (BMI), glycemia, liver enzymes, creatinine, or hemoglobin between PD and control (CT) groups (Table 1). Disease severity, medication status, gastrointestinal symptoms (constipation), and other potential clinical confounders are summarized in Table 1.

**Figure 1:**
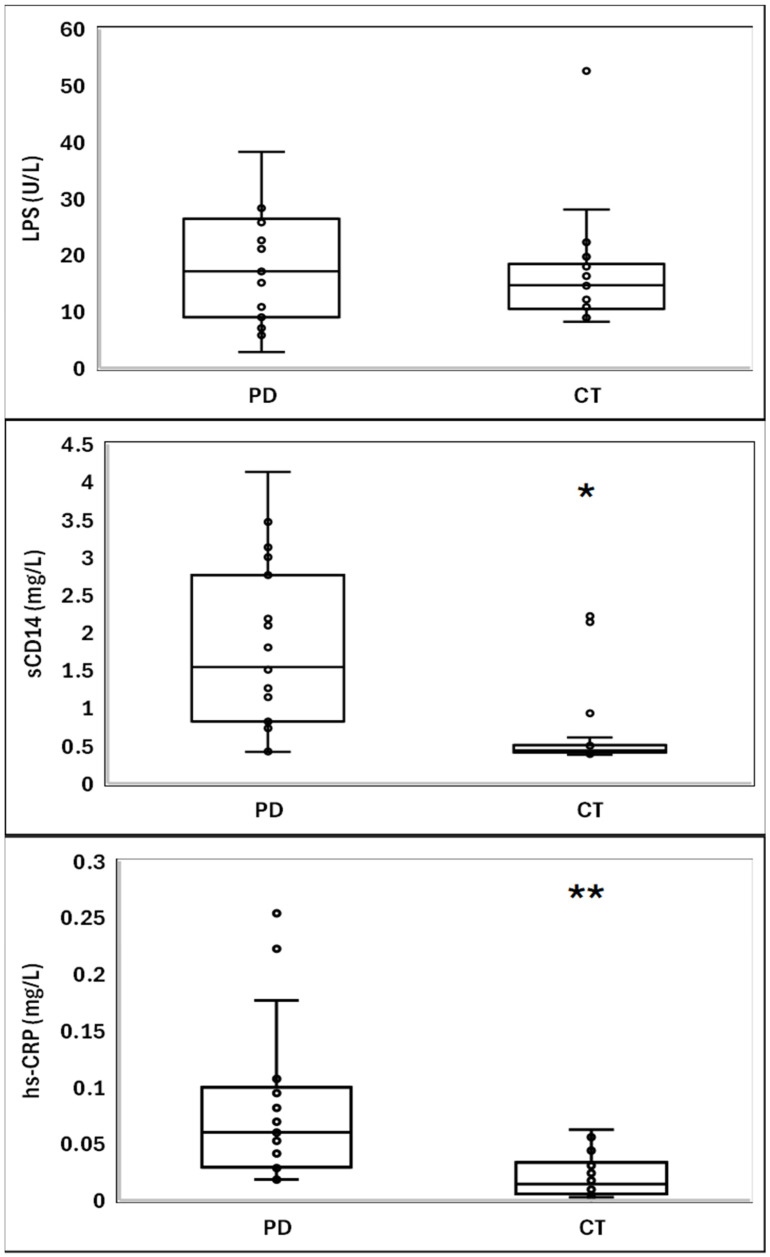
Plasma levels of lipopolysaccharide (LPS) and inflammatory markers in patients with Parkinson’s disease (PD) and healthy controls (CT). The center line denotes the median value (50th percentile), while the box contains the 25th to 75th percentiles of the dataset. The black whiskers mark the 5th and 95th percentiles, and values beyond these bounds are considered outliers. Statistical comparisons between groups were performed using the Mann–Whitney U test (two-tailed); significance is indicated as * *p* < 0.001 and ** *p* < 0.0001 versus CT. Abbreviations: LPS, lipopolysaccharide; sCD14, soluble cluster of differentiation 14; hs-CRP, high-sensitivity C-reactive protein.

**Figure 2:**
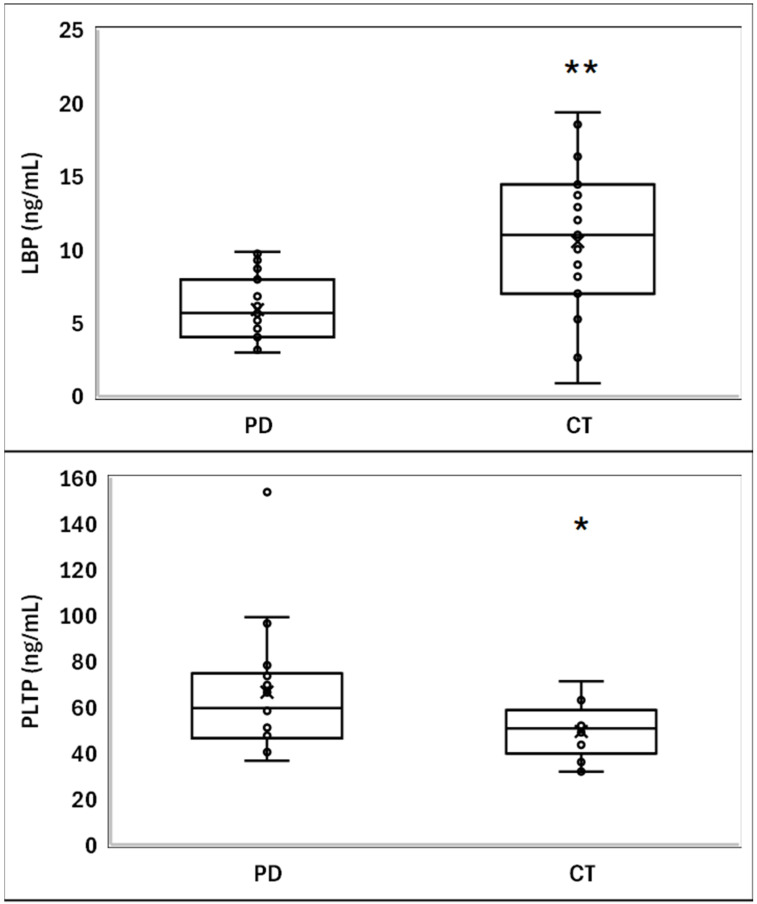
Plasma levels of lipopolysaccharide-binding protein and phospholipid transfer protein in patients with Parkinson’s disease (PD) and healthy controls (CT). The center line denotes the median value (50th percentile), while the box contains the 25th to 75th percentiles of the dataset. The black whiskers mark the 5th and 95th percentiles, and values beyond these bounds are considered outliers. Statistical comparisons between groups were performed using the Mann–Whitney U test (two-tailed); significance is indicated as * *p* < 0.05 and ** *p* < 0.001 versus CT. Abbreviations: LBP, lipopolysaccharide-binding protein; PLTP, phospholipid transfer protein.

Participants with PD exhibited a distinct inflammatory profile, characterized by significantly elevated concentrations of sCD14 and hsCRP compared with controls (Figure 1). Specifically, sCD14 levels were markedly higher in the PD group (1541 [1941] ng/mL) than in the CT group (438 [96] ng/mL), and hsCRP concentrations showed a similar pattern, increasing to 0.06 (0.07) mg/L in PD versus 0.01 (0.03) mg/L in CT. In contrast, circulating LPS levels did not differ significantly between groups (Figure 1). Regarding LPS-handling proteins, PD participants displayed substantially reduced LBP levels (5.68 [3.95] ng/mL) compared with controls (11.02 [7.44] ng/mL), whereas PLTP concentrations were significantly elevated in the PD group (59.58 [30.67] ng/mL) relative to the CT group (50.66 [13.34] ng/mL) (Figure 2).

Plasma lipid analysis revealed marked alterations in the PD group compared with controls (Table 2). Total cholesterol concentrations were significantly lower in individuals with PD (184 [51] mg/dL) than in the CT group (208 [36] mg/dL), and a similar reduction was observed for HDL-C (46 [12] mg/dL vs. 71 [21] mg/dL) and total PLs (184 [37] mg/dL vs. 208 [32] mg/dL). HDL-associated PLs were likewise decreased in the PD group (25 [10] mg/dL) relative to controls (35 [12] mg/dL). In contrast, triacylglycerol (TAG) levels did not differ significantly between the two groups.

Correlation analyses suggested a structured pattern of associations between inflammatory mediators and lipid fractions, highlighting coordinated alterations in immune–lipid interplay across the cohort (Table 3, Table 4 and Table 5).

In the overall population (Table 3), LPS concentrations were positively correlated with both sCD14 and PLTP. Additional positive correlations were identified between LBP and HDL-C, sCD14 and hsCRP, and hsCRP and PLTP. PLs, total cholesterol, and HDL-associated PLs all showed strong positive associations with HDL-C. In contrast, inverse correlations emerged between LPS and PLs or HDL-C, between LBP and sCD14, and between sCD14 and HDL-C. HsCRP also displayed negative associations with PL and HDL-C, while PLTP levels correlated negatively with PLs. TAGs exhibited inverse correlations with both HDL-PL and HDL-C.

Subgroup-specific correlation analyses revealed distinct patterns between PD patients and controls (Table 4 and Table 5). In the PD group (Table 4), positive correlations were observed between LPS and LBP, LBP and PLTP, sCD14 and hsCRP, PLTP and PLs, PLs and TAGs, and between HDL-associated phospholipids and HDL-C. Negative correlations were identified between PLTP and PLs, and between TAGs and HDL-associated phospholipids or HDL-C. In the control group (Table 5), positive correlations were limited to those between sCD14 and HDL-associated phospholipids and between hsCRP and HDL-C, whereas inverse correlations were observed between LPS and both sCD14 and hsCRP.

## 3. Discussion

In this study, we characterized associations among circulating inflammatory markers related to endotoxin handling and plasma lipid profiles in patients with Parkinson’s disease (PD) compared with matched controls, including increased sCD14 and hsCRP, reduced LBP, and elevated PLTP, together with lower total cholesterol and PLs, HDL-C, and HDL- PLs, but unchanged TAGs. Circulating LPS did not differ between groups. Correlation analyses suggested associations between inflammatory mediators and lipid fractions, with disease-specific interaction patterns in PD versus controls. Given the cross-sectional and observational design, these findings should be interpreted as associative and hypothesis-generating rather than as evidence of causality.

PD is known to exhibit a broad prodromal phase involving gastrointestinal dysfunction, constipation, and microbiome alterations that indicate impaired epithelial barrier integrity, mucosal inflammation, and increased exposure to gut-derived microbial products. Reviews by Hirayama et al. [18] and Zhang et al. [19] summarize evidence for gut dysbiosis in PD, including Akkermansia overrepresentation, reduced short-chain-fatty-acid-producing taxa, and potential vagal propagation mechanisms. Experimental work has further demonstrated the presence of enteric HDL3–LBP complexes capable of masking LPS from TLR4 activation and directing it toward enzymatic inactivation, which constitutes a mechanism of “disease tolerance” at the gut–portal–liver interface [20] that is conceptually extensible to PD’s systemic milieu. Finally, evidence from prospective studies shows that increased pre-diagnostic levels of LBP—a biomarker of chronic low-grade endotoxemia—are associated with a higher future risk of PD, with stronger associations in women and individuals with higher BMI, suggesting early systemic exposure to gut-derived LPS [21]. These observations have led to the hypothesis that altered host–microbe interactions may influence systemic inflammatory tone in PD; however, the extent to which circulating biomarkers reflect local intestinal or tissue-specific processes remains uncertain.

The marked rise in sCD14 and hsCRP in PD, in the absence of group differences in circulating LPS, is consistent with enhanced innate immune activation. sCD14 reflects monocyte–macrophage activation and CD14-dependent signaling, while hsCRP is a marker of low-grade systemic inflammation. However, the dissociation between unchanged circulating LPS levels and altered downstream markers remains speculative. Circulating LPS measurements are subject to substantial temporal variability and analytical limitations and may not capture episodic, localized, or compartmentalized endotoxin exposure. Therefore, elevated sCD14 and hsCRP may reflect cumulative or indirect immune activation rather than contemporaneous increases in plasma LPS.

Alterations in LBP and PLTP levels observed in PD may reflect changes in hepatic production, consumption dynamics, redistribution, or regulation by inflammatory mediators, rather than direct evidence of impaired or enhanced endotoxin neutralization. LBP and PLTP extract LPS from bacterial membranes and transfer it to HDL for functional neutralization, a process central to dampening TLR4-driven signaling [15]. In PD, a primed inflammatory milieu, upregulated TLR pathways, and heightened monocytic reactivity to LPS have been documented [3,22]. While reduced LBP has been reported in PD and linked to gut inflammation [9,23], it remains unclear whether this represents a cause or a consequence of systemic immune activation. Similarly, elevated PLTP may represent a compensatory response aimed at maintaining LPS handling in the context of altered lipid availability, although alternative explanations cannot be excluded.

Profound reductions in HDL-C and PLs—both total and HDL-associated—were evident in PD. Because phospholipid-rich HDL surfaces enhance LPS sequestration and neutralization, quantitative and compositional deficits in HDL could undermine host detoxification capacity and shift signaling toward CD14/TLR4 pathways. This interpretation is consistent with prior evidence implicating lipid pathway dysfunction in PD [24,25,26] and with observations that phospholipid content critically shapes HDL’s anti-endotoxin actions [27,28]. Furthermore, converging clinical and systems-level data indicate pervasive lipid pathway disturbances in PD, positioning HDL quantity and quality as potential modifiers of peripheral inflammatory tone [29,30].

At the cohort level, the positive correlations observed between LPS and both sCD14 and PLTP, along with the correlation between hsCRP and PLTP, may indicate a coordinated activation of LPS-responsive and lipid-associated pathways in the context of systemic inflammatory pressure. At the same time, the inverse associations linking LPS with HDL-C and phospholipids could indicate that the efficiency of endotoxin detoxification is influenced by lipoprotein availability and surface lipid composition. Similarly, the negative correlations between TAGs and HDL-related parameters may point to a broader remodeling of lipid transport processes that accompanies inflammatory activation [31,32]. Subgroup analyses further refine this interpretation. In individuals with Parkinson’s disease, the concomitant LPS–LBP and LBP–PLTP correlations, together with the close association between sCD14 and hsCRP, are suggestive of an increased engagement of endotoxin-sensing and trafficking mechanisms. In parallel, the inverse relationships observed between PLTP and phospholipids, as well as between TAGs and HDL-associated metrics, may reflect a relative inefficiency or increased functional demand on HDL remodeling pathways under conditions of sustained inflammatory burden. In contrast, control subjects display a more regulated pattern of associations, including negative correlations between LPS and both sCD14 and hsCRP, which appears compatible with a more effective containment of endotoxin signaling and a restrained activation of innate immune responses. However, given the modest sample size and multiple testing, these correlations should be interpreted with caution and viewed as exploratory. While positive associations among LPS-related markers and inverse correlations between inflammatory indices and HDL-related parameters are consistent with coordinated immune–lipid interactions, they do not establish directionality or causality and may be influenced by unmeasured confounders [31,32].

Taken together, the present results describe an associative inflammatory and lipid profile in PD that is consistent with altered regulation of endotoxin-related immune pathways and lipid metabolism. Rather than indicating increased systemic endotoxemia, the data suggest that downstream mediators of innate immune activation and lipid availability may be altered in PD, potentially modulating inflammatory responses [16,19,20,22,23,24,33,34].

A key strength of this study is the exclusion of obese participants, which helped minimize the confounding influence of excess adiposity on inflammatory and lipid parameters. Furthermore, the use of life partners as controls provided close matching for environmental, lifestyle, and dietary factors, thereby reducing variability in systemic inflammatory and metabolic profiles and strengthening the robustness of the comparative analyses. Moreover, all assays were performed under strictly sterile, pyrogen-free conditions, thereby minimizing technical variability related to potential endotoxin contamination. Nonetheless, several limitations should be acknowledged in this study. Owing to the cross-sectional design, causal or temporal relationships between inflammatory markers, LPS-related proteins, and lipid alterations cannot be inferred, and the findings therefore reflect associations observed at a single time point. Moreover, the limited sample size reduces statistical power, particularly for subgroup analyses. Consequently, subgroup-specific correlations should be considered exploratory and require validation in larger, independent cohorts. Although sex distribution was balanced and controls were carefully matched, the study was not sufficiently powered to support reliable sex-stratified analyses or robust interaction testing. Future investigations involving larger populations will be necessary to examine potential sex-specific differences in inflammatory and lipid profiles in Parkinson’s disease. Furthermore, despite careful documentation of relevant clinical confounders, residual confounding cannot be fully excluded in this observational setting. All analyses were based on circulating biomarkers, which may not fully capture tissue-specific processes occurring in the gut, liver, or central nervous system. Notably, circulating LPS concentrations may not accurately reflect chronic endotoxin exposure or functional LPS-neutralization capacity. Collectively, these limitations underscore the need for larger, longitudinal, and mechanistic studies integrating functional lipid assays, microbiome profiling, and repeated clinical assessments to better elucidate the role of immune–lipid interactions in Parkinson’s disease.

## 4. Materials and Methods

Participants were recruited at the Istituti Clinici di Perfezionamento in Milan, Italy. The study included 20 patients with Parkinson’s disease (PD) and 20 age- and sex-matched controls (CT) who were the life partners of PD patients. Inclusion criteria specified that participants had to be over 18 years old and have a body mass index (BMI) between 18.5 and 29.9 kg/m^2^. Exclusion criteria included diabetes, gastrointestinal diseases, pregnancy, medications that affect metabolism, including lipid-lowering therapy, endocrine disorders, liver or kidney dysfunction, malignancies, substance abuse, psychiatric illnesses, and engaging in intensive physical activity. The PD subjects met the UK Brain Bank diagnostic criteria and included both treated and newly diagnosed cases. Disease severity was assessed using the Hoehn and Yahr scale [35]. Clinical staging was performed by an experienced neurologist during the OFF-medication state and used for descriptive purposes only. The clinical trial is registered on ClinicalTrials.gov with the following number: NCT03937284.

Fasting venous blood samples were obtained, processed, and subsequently stored at −80 °C for analysis. LPS concentrations were quantified utilizing the Limulus Amebocyte Lysate (LAL) assay (kinetic-QCL, Lonza, Milan, Italy). Additionally, levels of LBP, sCD14, hsCRP, and PLTP were measured using enzyme-linked immunosorbent assay (ELISA) kits sourced from RayBio (Peachtree Corners, GA, USA), ELK Biotechnology (Sugar Land, TX, USA), and Elabscience (Houston, TX, USA). Lipid extraction was performed in accordance with the Folch method [36], while PLs were quantified using the Bartlett assay [37]. HDL was isolated through the Lopes-Virella precipitation method [38].

All assays were performed using sterile, pyrogen-free plastic materials and sterilized glassware.

The variables did not meet the assumptions of normality; therefore, data are reported as median values with their corresponding interquartile ranges (IQR). In accordance, group differences were evaluated using the Mann–Whitney U test, and associations among inflammatory and lipid markers were examined through Spearman’s rank-order correlation. Correlation analyses were performed for exploratory purposes. No formal correction for multiple comparisons was applied.

## 5. Conclusions

This study describes an altered profile of inflammatory markers related to endotoxin sensing and plasma lipid parameters in Parkinson’s disease. Patients with PD exhibited higher circulating sCD14 and hsCRP levels, together with changes in LBP, PLTP, HDL-cholesterol, and phospholipid fractions, while circulating LPS concentrations were comparable to those of controls. These findings indicate an association between peripheral innate immune activation and lipid alterations in PD, rather than providing evidence of increased systemic endotoxemia or causal mechanisms.

The present results are derived from a cross-sectional analysis and should therefore be interpreted as hypothesis-generating. Altered relationships between inflammatory and lipid biomarkers, including subgroup-specific correlation patterns, require confirmation in larger and independent cohorts. Future longitudinal and mechanistic studies will be necessary to determine whether disruptions of immune–lipid homeostasis contribute to disease onset, progression, or symptom burden, and to assess whether targeting the gut–immune–lipid axis may hold translational relevance in Parkinson’s disease.

## Figures and Tables

**Table 1:** Main characteristics of the population.

	PD	CT
Age (years)	61 (17)	59 (7)
BMI (kg/m^2^)	26.23 (3.8)	24.30 (4.9)
Sex (M/F)	11/9	11/9
Glycemia (mg/dL)	86 (7)	88 (26)
AST (U/L)	15 (4)	18.5 (3)
ALT (U/L)	11 (9)	25.5 (9)
Creatinine	0.75 (0.23)	0.9 (014)
Hb	13.9 (2.1)	14.1 (1.0)
Disease duration (years)	10 (11)	
Hoehn and Yahr stage: Off	2.5 (1)	
Dopaminergic therapy (yes/no)	19/1	
Constipation (yes/no)	13/7	
Protein-redistribution diet (yes/no)	10/10	

Data are expressed as median (IQR); PD: Parkinson’s disease group; CT: control group; BMI: body mass index; ALT: alanine aminotransferase; AST: aspartate aminotransferase; Hb: hemoglobin.

**Table 2:** Plasma lipid levels in Parkinson’s disease patients (PD) and controls (CT).

	PD	CT	*p* *
Chol (mg/dL)	184 (51)	208 (36)	**0.00331**
TAGs (mg/dL)	91 (33)	77 (55)	0.9688
PLs (mg/dL)	184 (37)	208 (32)	**0.01085**
HDL-C (mg/dL)	46 (12)	71 (21)	**<0.00001**
HDL-PLs (mg/dL)	25 (10)	35 (12)	**0.01803**

* *p*-values of the Mann–Whitney test. Data are median (IQR); Chol: total cholesterol; TAGs: triacylglycerols; PLs: phospholipids; HDL-C: HDL cholesterol; HDL-PLs: HDL phospholipids. Values shown in bold indicate statistically significant results.

**Table 3:** Spearman correlations between inflammatory indices and plasma lipids in the population.

		LPS	LPB	sCD14	hsCRP	PLTP	PLs	Chol	TAGs	HDL-PL	HDL-C
		(EU/L)	(ng/mL)	(ng/mL)	(mg/L)	(ng/mL)	(mg/dL)	(mg/dL)	(mg/dL)	(mg/dL)	(mg/dL)
**LPS**	R	1.000	−0.278	0.327	0.300	0.424	−0.328	−0.212	0.012	−0.220	−0.479
(EU/L)	*p*	--	0.095	**0.048**	0.072	**0.012**	**0.048**	0.208	0.947	0.190	**0.003**
**LPB**	R	−0.278	1.000	−0.357	−0.217	−0.063	0.072	0.292	−0.179	0.260	0.414
(ng/mL)	*p*	0.095	--	**0.028**	0.190	0.718	0.668	0.075	0.295	0.115	**0.010**
**sCD14**	R	0.327	−0.357	1.000	0.578	0.013	−0.225	−0.225	0.124	−0.265	−0.365
(ng/mL)	*p*	**0.048**	**0.028**	--	**1.455 × 10^−4^**	0.942	0.174	0.174	0.471	0.107	**0.024**
**hsCRP**	R	0.300	−0.217	0.578	1.000	0.445	−0.390	−0.311	−0.091	−0.231	−0.343
(mg/L)	*p*	0.072	0.190	**1.455 × 10^−4^**	--	**0.007**	**0.015**	0.058	0.598	0.163	**0.035**
**PLTP**	R	0.424	−0.063	0.013	0.445	1.000	−0.508	−0.317	−0.180	−0.123	−0.234
(ng/mL)	*p*	**0.012**	0.718	0.942	**0.007**	--	**0.002**	0.063	0.317	0.482	0.177
**PLs**	R	−0.328	0.072	−0.225	−0.390	−0.508	1.000	0.214	0.271	0.254	0.433
(mg/dL)	*p*	**0.048**	0.668	0.174	**0.015**	**0.002**	--	0.196	0.110	0.125	**0.007**
**Chol**	R	−0.212	0.292	−0.225	−0.311	−0.317	0.214	1.000	−0.184	0.112	0.334
(mg/dL)	*p*	0.208	0.075	0.174	0.058	0.063	0.196	--	0.284	0.502	**0.041**
**TAGs**	R	0.012	−0.179	0.124	−0.091	−0.180	0.271	−0.184	1.000	−0.372	−0.393
(mg/dL)	*p*	0.947	0.295	0.471	0.598	0.317	0.110	0.284	--	**0.025**	**0.018**
**HDL-PL**	R	−0.220	0.260	−0.265	−0.231	−0.123	0.254	0.112	−0.372	1.000	0.584
(mg/dL)	*p*	0.190	0.115	0.107	0.163	0.482	0.125	0.502	**0.025**	--	**1.183 × 10^−4^**
**HDL-C**	R	−0.479	0.414	−0.365	−0.343	−0.234	0.433	0.334	−0.393	0.584	1.000
(mg/dL)	*p*	**0.003**	**0.010**	**0.024**	**0.035**	0.177	**0.007**	**0.041**	**0.018**	**1.183 × 10^−4^**	--

R: Spearman correlation; *p*: *p*-value; LPS: lipopolysaccharide; LBP: lipopolysaccharide binding protein; sCD14: soluble cluster of differentiation 14; hsCRP: high-sensitivity C-reactive protein; PLTP: phospholipid transfer protein; PLs: phospholipids; Chol: total cholesterol; TAGs: triacylglycerols; HDL-C: HDL cholesterol. Values shown in bold indicate statistically significant results.

**Table 4:** Spearman correlations between inflammatory indices and plasma lipids in Parkinson’s disease patients.

		LPS	LPB	sCD14	hsCRP	PLTP	PLs	Chol	TAGs	HDL-PL	HDL-C
		(EU/L)	(ng/mL)	(ng/mL)	(mg/L)	(ng/mL)	(mg/dL)	(mg/dL)	(mg/dL)	(mg/dL)	(mg/dL)
**LPS**	R	1.000	0.486	−0.250	−0.056	0.315	−0.076	0.213	0.073	0.031	0.022
(EU/L)	*p*	--	**0.035**	0.302	0.819	0.203	0.756	0.381	0.767	0.901	0.927
**LPB**	R	0.486	1.000	−0.189	0.346	0.505	−0.293	0.125	−0.079	0.109	−0.166
(ng/mL)	*p*	**0.035**	--	0.437	0.147	**0.033**	0.223	0.609	0.748	0.658	0.496
**sCD14**	R	−0.250	−0.189	1.000	0.582	−0.437	0.104	0.168	−0.105	−0.153	−0.009
(ng/mL)	*p*	0.302	0.437	--	**0.009**	0.070	0.673	0.493	0.668	0.533	0.971
**hsCRP**	R	−0.056	0.346	0.582	1.000	0.051	−0.151	−0.022	−0.132	0.012	0.075
(mg/L)	*p*	0.819	0.147	**0.009**	--	0.842	0.538	0.929	0.591	0.960	0.761
**PLTP**	R	0.315	0.505	−0.437	0.051	1.000	−0.478	−0.044	−0.348	0.218	0.039
(ng/mL)	*p*	0.203	**0.033**	0.070	0.842	--	**0.045**	0.861	0.157	0.385	0.877
**PLs**	R	−0.076	−0.293	0.104	−0.151	−0.478	1.000	−0.111	0.493	0.051	0.202
(mg/dL)	*p*	0.756	0.223	0.673	0.538	**0.045**	--	0.650	**0.032**	0.836	0.408
**Chol**	R	0.213	0.125	0.168	−0.022	−0.044	−0.111	1.000	−0.001	−0.226	−0.120
(mg/dL)	*p*	0.381	0.609	0.493	0.929	0.861	0.650	--	0.997	0.353	0.624
**TAGs**	R	0.073	−0.079	−0.105	−0.132	−0.348	0.493	−0.001	1.000	−0.516	−0.491
(mg/dL)	*p*	0.767	0.748	0.668	0.591	0.157	**0.032**	0.997	--	**0.024**	**0.033**
**HDL-PLs**	R	0.031	0.109	−0.153	0.012	0.218	0.051	−0.226	−0.516	1.000	0.850
(mg/dL)	*p*	0.901	0.658	0.533	0.960	0.385	0.836	0.353	**0.024**	--	**<0.0001**
**HDL-C**	R	0.022	−0.166	−0.009	0.075	0.039	0.202	−0.120	−0.491	0.850	1.000
(mg/dL)	*p*	0.927	0.496	0.971	0.761	0.877	0.408	0.624	**0.033**	**<0.0001**	--

R: Spearman correlation; *p*: *p*-value; LPS: lipopolysaccharide; LBP: lipopolysaccharide binding protein; sCD14: soluble cluster of differentiation 14; hsCRP: high-sensitivity C-reactive protein; PLTP: phospholipid transfer protein; PLs: phospholipids; Chol: total cholesterol; TAGs: triacylglycerols; HDL-C: HDL cholesterol. Values shown in bold indicate statistically significant results.

**Table 5:** Spearman correlations between inflammatory indices and plasma lipids in control subjects.

		LPS	LPB	sCD14	hsCRP	PLTP	PLs	Chol	TAGs	HDL-PL	HDL-C
		(EU/L)	(ng/mL)	(ng/mL)	(mg/L)	(ng/mL)	(mg/dL)	(mg/dL)	(mg/dL)	(mg/dL)	(mg/dL)
**LPS**	R	1	0.030	−0.544	−0.573	−0.300	−0.249	0.033	−0.202	−0.150	−0.222
(EU/L)	*p*	--	0.906	**0.020**	**0.013**	0.259	0.320	0.896	0.454	0.553	0.377
**LPB**	R	0.030	1.000	0.244	−0.158	−0.056	−0.139	0.103	−0.121	0.110	0.191
(ng/mL)	*p*	0.906	--	0.314	0.519	0.830	0.571	0.676	0.642	0.655	0.435
**sCD14**	R	−0.544	0.244	1.000	0.307	0.130	0.011	−0.284	0.058	0.505	0.069
(ng/mL)	*p*	**0.020**	0.314	--	0.201	0.619	0.966	0.238	0.826	**0.028**	0.780
**hsCRP**	R	−0.573	−0.158	0.307	1.000	−0.110	0.186	−0.119	0.110	0.252	0.463
(mg/L)	*p*	**0.013**	0.519	0.201	--	0.673	0.446	0.626	0.673	0.298	**0.046**
**PLTP**	R	−0.300	−0.056	0.130	−0.110	1.000	0.039	0.349	0.018	−0.118	0.392
(ng/mL)	*p*	0.259	0.830	0.619	0.673	--	0.881	0.169	0.950	0.653	0.119
**PLs**	R	−0.249	−0.139	0.011	0.186	0.039	1.000	0.225	0.369	0.196	0.071
(mg/dL)	*p*	0.320	0.571	0.966	0.446	0.881	--	0.355	0.145	0.422	0.771
**Chol**	R	0.033	0.103	−0.284	−0.119	0.349	0.225	1.000	0.460	−0.048	0.247
(mg/dL)	*p*	0.896	0.676	0.238	0.626	0.169	0.355	--	0.063	0.844	0.307
**TAGs**	R	−0.202	−0.121	0.058	0.110	0.018	0.369	0.460	1.000	0.368	−0.219
(mg/dL)	*p*	0.454	0.642	0.826	0.673	0.950	0.145	0.063	--	0.146	0.399
**HDL-PLs**	R	−0.150	0.110	0.505	0.252	−0.118	0.196	−0.048	0.368	1.000	0.074
(mg/dL)	*p*	0.553	0.655	**0.028**	0.298	0.653	0.422	0.844	0.146	--	0.764
**HDL-C**	R	−0.222	0.191	0.069	0.463	0.392	0.071	0.247	−0.219	0.074	1.000
(mg/dL)	*p*	0.377	0.435	0.780	**0.046**	0.119	0.771	0.307	0.399	0.764	--

R: Spearman correlation; *p*: *p*-value; LPS: lipopolysaccharide; LBP: lipopolysaccharide binding protein; sCD14: soluble cluster of differentiation 14; hsCRP: high-sensitivity C-reactive protein; PLTP: phospholipid transfer protein; PLs: phospholipids; Chol: total cholesterol; TAGs: triacylglycerols; HDL-C: HDL cholesterol. Values shown in bold indicate statistically significant results.

## Data Availability

Data are not available for privacy/ethical reasons.

## Abbreviations

The following abbreviations are used in this manuscript:

α-Syn	α-Synuclein
ALT	Alanine Aminotransferase
AST	Aspartate Aminotransferase
BMI	Body Mass Index
Chol	Cholesterol
CD	Cluster Of Differentiation
hsCRP	High-Sensitivity C-Reactive Protein
CT	Control Group
ELISA	Enzyme-Linked Immunosorbent Assay
Hb	Hemoglobin.
HDL	High-Density Lipoprotein
HDL-C	High-Density Lipoprotein Cholesterol
LAL	Limulus Amebocyte Lysate
LBP	Lipopolysaccharide-Binding Protein
LPS	Lipopolysaccharide
PD	Parkinson’s Disease
PLs	Phospholipids
PLTP	Phospholipid Transfer Protein
TAGs	Triacylglycerols
TLR	Toll-Like Receptor
